# The role of inhibition capacities in the Iowa gambling test performance in young tattooed women

**DOI:** 10.1186/s40359-019-0363-3

**Published:** 2019-12-23

**Authors:** Semion Kertzman, Alex Kagan, Omer Hegedish, Rina Lapidus, Abraham Weizman

**Affiliations:** 1 Beer-Ya’akov-Ness Ziona Mental Health Center, Forensic Psychiatry Division, Ness Ziona, Israel; 2 Sackler Faculty of Medicine, Tel Aviv University, Tel Aviv, Israel; 3 The Program for Hermeneutics and Cultural Studies, Interdisciplinary Studies Unit, Bar-Ilan University, Ramat Gan, Israel; 4 Department of Neuro-Pathopsychology, L.S. Vygotsky Institute of Psychology, RSUH, Moscow, Russia; 5 Ashkelon Academic College, Ashkelon, Israel; 6 Department of Psychology, University of Haifa, Haifa, Israel; 7 Comparative Literature Department, Bar-Ilan University, Ramat Gan, Israel; 8 Research Unit, Geha Mental Health Center and Felsenstein Medical Research Center, Petah Tikva, Israel

**Keywords:** Tattoo, IGT, Inhibition, Impulsivity

## Abstract

**Background:**

Using the Iowa Gambling Test (IGT), we demonstrated previously impaired decision- making process in young tattooed women. The purpose of the present study was to explore the associations among the three facets of impaired inhibition (response inhibition, reflection inhibition and interference inhibition) and decision-making processes in this population.

**Methods:**

To this end, the participants of the previous study (60 tattooed women and 60 non-tattooed women) were assessed in the Go/NoGo task, a measure of response inhibition, the Matched Familiar Figure Test (MFFT), a measure of reflection inhibition and the Stroop task a measure of interference inhibition.

**Results:**

Tattooed women were significantly slower than non-tattooed women in the Go/NoGo performance; however, no differences were detected in the MFFT and the Stroop task. A hierarchical regression analysis did not reveal any significant main effects of these inhibition measures on the IGT performance.

**Conclusions:**

These findings do not support the hypothesis that risky decision in young tattooed women is due to impaired inhibitory control. Further studies are needed to identify the cognitive mechanisms involved in the tendency to risky decisions in young tattooed women.

## Background

Young people are likely to engage in risky behaviors, such as drinking alcohol, taking illegal drugs, having unprotected sex, engaging in delinquent activity, and driving recklessly [1]. Empirical research found association between getting tattoos and higher frequency of these behaviors, as well as, to engage in illegal activities, problem gambling, dropping out of school, suicidal attempts, violence and death by homicide [2–4]. It possible that young people, who have tattoos is more open to engaging in risk-behavior [5–16] Although, it was previously shown that young individuals with tattoo display worse performance in decision-making tasks such the Iowa Gambling Task (IGT) [17] and elevated self-assessed impulsiveness [17, 18], the relation between the constructs in tattooed population was not analyzed. The decision making is a complex process, and understanding the role of more detailed cognitive processes such inhibition abilities responsible for risky decisions remains unclear. Findings from previous studies on this issue in non-tattooed samples were inconsistent. The mechanisms of risky behavior may be clarified by analysis of the interactions between decision making and inhibition abilities [19]. However, research on the association between decision making and inhibition abilities is inconsistent. Some studies claim that the inhibition abilities is an integral part of decision-making process and is aimed to protect a decision process from disruption by competing events of information streams [20–22] In addition, inhibition training was proved to be effective in reducing decision making errors [23]. In contrast, other studies suggest that decision making process is not in close association with inhibition abilities and propose complex interactions between biases, reasons, emotions, goals, motivations, competing resources and opportunities afforded by the social context [24, 25]. Moreover, some authors found that inhibition capacity can be dissociated from risky decision [26–31]. For example, it was found that excessive internet users have deficits in decision-making function, which are characterized by a strategy learning lag rather than an inability to learn from a task contingencies and their risky decisions are not related to response inhibition abilities [32]. Furthermore, it was demonstrated that sleep deprivation diminished initial pre-potent inhibition but not the decision making process [33]. An animal study also demonstrated that inhibition and risk taking decision are separate processes [34]..

Thus, it is unclear whether tattooing behavior can be independently predicted by a weak inhibitory control or risky decisions or both. The current study attempts to answer two main issues:

1. Are tattooed women exhibit risky behavior as a result of impaired inhibition capacities? It should be noted that inhibition is a complex concept. Bari and Robbins [35] suggested dividing inhibitory control into two categories: behavioral or *response inhibition* and cognitive inhibition or *resistance to distractor interference*. In addition, Kagan [36] introduced the concept of *reflection inhibition* as a capacity to choose the correct course of action from a number of possibilities prolonging the time before action is taken. We assessed these three inhibition capacities in an attempt to identify which inhibition facet is associated with getting tattoo.
2. Is there an effect of inhibition capacities on decision making process in young women with tattoo? It is expected that in tattooed population there is an association between risky decision and limited inhibition resources. The current study attempted to analyze influence of different inhibition mechanisms on the IGT performance in a sample of tattooed young women assumed to exhibit high level of impulsivity trait compared to a non-tattooed population [17, 18, 37, 38]. We assumed that risky decisions are associated differently with inhibition capacities in women with high impulsivity (tattooed) compared to those with low impulsivity (non-tattooed controls).

To our knowledge, there are no studies on the evaluation of inhibition capacities among tattooed population and the relation between decision-making process and inhibition capacities in women with tattoos.

## Methods

As described in previous publications [17, 39] the entire research process took five months (March–July 2012), and included locating candidates for the study in the Tel Aviv area, through advertising at universities, in social networks (Facebook) and using personal contacts. Women (research and control), with and without tattoos, from similar socio economic backgrounds (employed, students or graduates) were invited to apply to be part of a research project authorized by Bar-Ilan University Review Board (Ramat Gan, Israel) focusing on the decision making process of both groups. Prior to being accepted to the study, all participants underwent an evaluation to determine their eligibility, which included questions on medical history, illicit drug use, family and personal psychiatric history. All were free of any psychopharmacologic treatment. The 120 women (who were also part of our study on the association between tattooing behavior and risky decisions as reflected in IGT) signed a consent form to be part of a free study and in return receive a complimentary consultation regarding their inhibition capacity and professional guidance about their neurocognitive and personality assessments. The individual sessions (up to 90 min each), also included a detailed description of the research aims.

The link between risky decision and inhibition capacities was analyzed using three laboratory measures.

A semi-structured interview [17] with a 20-item measure of tattoo characteristics was conducted by a researcher (AK) for 60 tattooed women (no men to prevent sex differences on the cognitive measures, or women who had removed tattoos) aged 18–35 (M = 28.4, SD = 5.95), with no neurological disorders, mental retardation, and no record of substance abuse/dependence (other than smoking). All were employed or students, 58% had more than one tattoo, all were employed or students: high school diploma or lower 46.7%, bachelor’s degree: 25%, master’s degree 23.3% and philosophy: 5%. Of this entire group of tattooed women, 55% were smokers.

The 60 non-tattooed women (control group) did not include anyone with current or past DSM-IV-TR axis I psychiatric disorder. Participants were of similar ages, 18–35 (M = 28.5, SD = 5.43). The education level for this group was as follows: high school diploma or lower – 25%, first university degree – 28.3%, second university degree – 41.7% and philosophy degree – 5%. The percentage of smokers in this group was only 10%.

We used one decision-making measure (IGT) and three inhibition measures (Go/NoGo task, MFFT, Stroop task) for our analysis. The IGT was used in our previous study [17].

### Measurements

#### Decision making measure: computerized animation variant of the Iowa gambling test

As a simulation of real-life decision-making, the IGT involves weighing expected, but uncertain, rewards and penalties (for review [40]). Diminished performance on the IGT expresses the participant’s failing to learn from punishment cues, and ability to improve their decisions in the face of changing contingencies [40]. The IGT is a favored measure available to gauge risky decisions [22]. This test is presented in the form of a game (we used “Casino” AnimaScan Ltd., Ashdod, Israel, 2000 a computerized animation version of IGT [41]).

The IGT requires choosing between several different alternatives. Each trial has four decks of cards to choose from. The four decks of cards A, B, C, D were placed one beside the other simultaneously on a computer screen. Participants knew that teach deck gives them virtual money, however, they were unaware that A and B were termed risky, and their long-term results are negative, while C and D were safe decks with a more positive overall outcome. They gains were also varied. Participants were instructed that they had 100 options to choose from and their aim was to earn as much virtual money as possible. The choices (selection of cards from different decks) are either advantageous or disadvantageous, but each choice is associated with ambiguity regarding the outcome, since it is difficult for the subject to keep track and remember the gains and losses from previous trials [42]. In the beginning, the IGT simulates a specific situation “decisions under ambiguity”. This means that subjects have no information about the choices’ consequences and probabilities. Later, by using the feedback of previous choices, subjects can learn the rules (most likely after the first half of 100 trials), and, thus, the IGT assesses “decisions under risk.” [42] Participants play the game several times and get results, and learn to choose the safe card decks over the risky ones. A ‘net score’ was calculated for the each participant according to deck selections [(A + B)-(C + D)] [42]. Although the IGT’s sensitivity for detecting decision-making impairment is well established, recent studies have highlighted the complexity of this task [28, 43, 44].

#### Inhibition measures: the go/NoGo task

Response inhibition can be defined as a cognitive action that enables a person to repress certain behaviors or reactions. The purpose of the Go/NoGo task is to estimate whether a behavior that is not appropriate can be controlled [45]. In this 5-min test, participants were given 120 red rectangles (‘Go’) and 30 black rectangles (‘NoGo’) and asked to complete 150 trials. Stimulus was presented in random order at a rate of one stimulus per 2000 ms and participants were told to respond to the stimuli with a Go or withhold response NoGo. A constant inter-stimulus interval was present for minimizing any orienting response caused by the unpredictability of a stimulus display. The Go/NoGo mean of response time (as sum of mean responses time of correct responses during the two blocks of the Go/NoGo task) was a measure of response inhibition ability. We applied a computerized variant of the Go/NoGo task (AnimaScan Ltd., Ashdod, Israel 2000) as previously described [46].

#### MFFT

The Matching Familiar Figures Task (MFFT) [36] was conducted in a computerized version [47] (AnimaScan Ltd., Ashdod, Israel 2000). Participants were directed to select the one (of six) possibility that matched the initial image. The following parameters were used: 1. Response time in milliseconds; 2. Number of errors committed during task.

#### The Stroop task

We use a manual key-press variant of the Stroop task [48] with unlimited time of stimulus presentation [49]. Subjects who are prone to impulsive behavior are expected to exhibit a weaker interference control, as proposed previously, [27, 50]. Participants were asked to read words and disregard the four font colors for words (green, red blue or yellow). The word is centered on a screen of a gray background printed in one of five colors: red, blue, green, yellow and black and is located above two colored rectangles on each side. Participants must press one of the two keys. The color of the word is always the same color of one of the rectangles and the other color is the meaning of the word. There were 40 ‘Neutral’ trials where the letters of the words were black, 40 ‘Congruent’ trials where the meaning of the word and the color of the letters corresponded and 40 ‘Incongruent’ trials where the meaning of the word did not correspond to the colors of the letter (word BLUE written in red).

#### Statistical analysis

Data were analyzed using SPSS (v. 19) software for Windows. All analyses used two-tailed levels of significance. The parametric (*t*-test) and non-parametric (*χ*^*2*^) tests were performed to compare group differences in demographic and behavioral parameters. For evaluation of differences between groups in the inhibition performance, the multivariate analysis of covariance (MANCOVA) was conducted with performance variables (mean of response time, variability of response time, numbers of errors, et ctr) as dependent measures and group (tattooed women and controls) as between-subject measures, with education and smoking as covariates. A hierarchical regression was conducted to examinee if variables of our interest: (i) Stroop interference reaction time (response time in the incongruent condition minus response time in the congruent condition), (ii) the Go/NoGo mean of response time (as sum of mean responses time in the blocks of the Go/NoGo task), and (iii) the total number of errors in the MFFT explain a statistically significant amount of variance in the IGT performance (sum of a net score for the 40 last selections: Trials 60–100). Since participants with high impulsivity perform poorly in the IGT due to a robust learning component [42], we separated early and late IGT selections and used only the 40 last selections and combined them into a single measure. This analysis allows correction of the significance levels for multiple tests when two or more response variables are tested from the same set of individuals.

## Results

Women with tattoos were significantly less educated (14.53 ± 2.77 versus 15.82 ± 2.63 in non-tattooed women; t = 2.60, df = 118, *p* = 0.01) and with a higher of rate of smoking status (55% versus 10%; χ2 = 27.69, df = 1, *p* < 0.0001) than non-tattooed women. No differences in age were found between the two groups (28.47 ± 5.42 versus 28.35 ± 5.95 years; t = 0.11, df = 118, *p* = 0.91). Thus education and smoking habit were considered as covariates.

The inhibition characteristics of the tattooed and non-tattooed women are shown in Tables 1, 2 and 3. Significant between-group differences were not found for both MFFT and Stroop tasks in terms of response time, variability of response time and number of errors. However, the tattooed women performed significantly more slowly than non-tattooed women in the Go/NoGo task, indicating impaired response inhibition.

**Table 1:** Multivariate Analysis of Covariance (MANCOVA) on the GoNoGo task

GoNoGo	Adjusted mean (SD)		F	P
	Tattooed	Control		
Overall				
Group			2.23^1^	.03*
Smoking			2.53^1^	.01*
Education			1.02^1^	.42
RT (part 1)				
Group	392.45 (8.18)	57.86 (8.118)	7.89	.00*
Smoking			2.20	.14
Education			2.86	.09
SD of RT (part 1)				
Group	75.14 (4.66)	72.22 (4.66)	.17	.67
Smoking			.14	.70
Education			.25	.61
Error of Omission (part 1)				
Group	.49 (1.18)	.52 (1.18)	.00	.93
Smoking			.39	.52
Education			.88	.34
Error of Commission (part 1)				
Group	.75 (.16)	1.14 (.16)	2.40	.12
Smoking			2.41	.12
Education			1.57	.21
RT (part 2)				
Group	393.09 (8.47)	45.40 (8.47)	13.97	.00***
Smoking			8.47	.00**
Education			2.73	.10
SD of RT (part 2)				
Group	83.28 (5.03)	66.14 (5.03)	5.12	.02*
Smoking			4.71	.03*
Education			.46	.49
Error of Omission (part 2)				
Group	.44 (.16)	.39 (.16)	.03	.84
Smoking			.25	.61
Education			1.16	.28
Error of Commission (part 2)				
Group	.97 (.019)	1.49 (.019)	3.08	.08
Smoking			4.03	.03*
Education		2.79		.09

Note. ^1^ Wilks’ Lambda F. Group = tattooed vs. non-tattooed women. *p < .05. **p < .01. *** *p* < .001. The means were adjusted by education and smoking habit that were considered as covariates. It is of note that in our previous study (Kertzman et al., 2015) smoking was considered as between-subject factor in repeated measures analysis of covariance (ANCOVA)

**Table 2:** Analysis of Covariance (MANCOVA) on Matching Familiar Figures Test (MFFT)

MFFT	Adjusted mean (SD)		F	P
	Tattooed	Control		
Overall				
Group			.57^1^	.74
Smoking			1.06^1^	.38
Education			1.57^1^	.16
First RT				
Group	16.27 (1.23)	14.84 (1.23)	.59	.44
Smoking			.49	.48
Education			3.85	.05
Mean RT				
Group	20.90 (1.33)	19.10 (1.33)	.80	.37
Smoking			3.73	.05
Education			6.05	.01
SD RT				
Group	13.55 (1.07)	12.01 (1.07)	.89	.34
Smoking			5.27	.02
Education			5.75	.01
Error				
Group	3.81 (.39)	3.90 (.39)	.02	.88
Smoking			1.37	.24
Education			1.08	.30
Error RT				
Group	20.98 (2.29)	23.47 (2.29)	.52	.47
Smoking			.27	.60
Education			.37	.54
Error SD				
Group	7.39 (1.09)	8.94 (1.09)	.89	.34
Smoking			.25	.61
Education			.75	.38

**Table 3:** Analysis of Covariance (MANCOVA) on Stroop test

Stroop	Adjusted mean		F	P
	Tattooed	Control		
Overall				
Group			28^1^	.97
Smoking			1.11^1^	.36
Education			1.40^1^	.19
Neutral RT				
Group	1031.40 (25.33)	1049.92 (25.33)	.23	.62
Smoking			.52	.47
Education			.14	.70
Neutral SD				
Group	299.24 (17.19)	297.00 (17.19)	.00	.93
Smoking			1.28	.26
Education			.01	.91
Neutral Errors				
Group	.17 (.07)	.27 (.07)	.69	.40
Smoking			3.42	.06
Education			.08	.76
Congruent RT				
Group	926.80 (24.78)	952.49 (24.78)	.47	.49
Smoking			1.01	.31
Education			.05	.81
Congruent SD				
Group	273.04 (20.40)	282.28 (20.40)	.09	.76
Smoking			1.78	.18
Education			.32	.57
Congruent Errors				
Group	.12 (.06)	.21 (.06)	.76	.38
Smoking			1.02	.31
Education			3.28	.07
Incongruent RT				
Group	1147.70 (28.12)	1164.86 (28.12)	.16	.68
Smoking			1.26	.26
Education			.67	.41
Incongruent SD				
Group	334.84 (18.76)	329.18 (18.76)	.04	.84
Smoking			.17	.67
Education			.36	.54
Incongruent Errors				
Group	1.97 (.36)	2.29 (.36)	.34	.56
Smoking			1.06	.30
Education			94	.33

Note. ^1^ Wilks’ Lambda F. Group = tattooed vs. non-tattooed women. * p < .05. **p < .01. *** p < .001

The effect of three inhibition measures on risky performance in the IGT was evaluated by the hierarchical regression analysis. The net score of the last two trials of the IGT (40 trials) was considered as the dependent variable. Three inhibition measures: (i) response inhibition (as sum of response time in the two blocks of the Go/NoGo task), (ii) the total number of errors in the MFFT and (iii) Interference Index (Response Time of Incongruence Condition – Response time of Congruent Condition) in the Stroop task were considered as independent measures. The hierarchical regression analysis did not reveal significant main effects of any inhibition measures on the risky decision process (Table 4).

**Table 4:** Summary of hierarchical linear regression analysis for variables predicting net-score in the IGT

	Model 1			Model 2			Model 3		
Variable	*B*	*SE B*	*β*	*B*	*SE B*	*β*	*B*	*SE B*	*β*
GoNoGo									
Education	.69	1.40	.04	.15	1.36	.01	.24	1.40	.01
Smoking	−2.52	2.81	−.08	2.64	3.05	.09	2.74	3.09	.09
Tattoo				−10.08	2.83	−.37**	−15.26	18.80	−.56
Sum RT				.00	.00	.12	.00	.00	.09
Tattoo X Sum RT							.00	.01	.20
*R*^*2*^	.01			.11			.11		
*F* for change in *R*^*2*^	.75			6.37**			.07		
MFFT									
Education				.39	1.36	.02	.56	1.36	.03
Smoking				1.92	3.04	.06	1.25	3.08	.04
Tattoo				−8.95	2.73	−.33**	−12.89	4.09	−.47**
Error				−.05	.42	−.01	−.65	−.63	−.13
Tattoo X Error							1.12	.86	.24
*R*^*2*^				.09			.11		
*F* for change in *R*^*2*^				5.35**			1.67		
Stroop									
Education				.21	1.36	.01	.38	1.33	.02
Smoking				1.7	3.01	.05	1.77	3.02	.06
Tattoo				−8.82	2.72	−.32**	−13.58	6.30	−.50*
Interference RT				−.01	.01	−.10	−.02	.01	−.17
Tattoo X									
Interference RT							.02	.02	.21
*R*^*2*^				.10			.11		
*F* for change in *R*^*2*^				6.04**			.70		

Note. *p < .05. **p < .01

## Discussion

Although impulsivity explains the relation between getting tattoo and increased risk- taking behavior, surprisingly this notion has received little empirical support. In contrast to our study, previous impulsivity studies in tattoo populations relied solely on self-report measures. The advantage of the current study is the use of three inhibition measures in order to clarify the contribution of several facets of risky behavior. The mechanisms involved in risky behavior in young non-criminal tattooed persons are not clear. Association between inhibition abilities and decision-making process is an important issue and its investigation can shed a light on the self-control in tattooed population.

The first main hypothesis of our current study was that tattooed women would exhibit risky behavior as a result of impaired inhibition capacities. Inhibition impairments were expected in tattooed population more frequently than in non-tattooed population since tattooing behavior was reported to be associated with wide range of risk taking behaviors and substance use disorders (see introduction). Analysis of the different inhibition processes may lead to a better understanding of risky behavior. Tattooed women did not display deficits in tasks measured reflexive inhibition and interference control, but impaired response inhibition as measured by the slow response time in the Go/NoGo task. In the Go/NoGo task, response slowness was a sensitive measure of inhibition impairment [51]. Impairment in the Go/NoGo performance, but not in the MFFT and the Stroop tasks performance, indicates that tattooed women show inhibition impairments in situations under time pressure only.

The second hypothesis of the current study was that tattooed women would exhibit risky decisions as a result of impaired inhibition capacity. Impulsive responses (faster with more errors) reflect reduced behavior monitoring efficiency and inhibition control that are expected when greater reward sensitivity is required [52]. In our study we did not find an association between fast-error performance on inhibition task and risky decision on the IGT. Contrary to our prediction, the hierarchical regression analysis did not reveal any significant main effects of the three inhibition measures on the IGT performance. Bechara [53] suggested that in contrast to a learned inhibition of a pre-potent response decision-making process involves an evaluation of the pros and cons of a given response options. A recent meta-analysis showed that healthy populations are not unequivocally good decision makers [54] and that a more than 30% of “normal” controls perform poorly on the IGT [55]. It is possible that the IGT (as measure of risky process of decision) may be a multi-dimensional task requiring several processes, including reversal learning, response inhibition, risk-seeking and deficits in strategic planning, cognitive biases, and hypersensitivity to reward [56]. It seems that the IGT assesses behavior in a fashion that combines multiple cognitive functions [57]. Executive functioning and working memory skills are important components of IGT performance, even in those without clinical disorders or evidence of “real world” dysfunction [55]. Moreover, the IGT reflects also an affective component of risk-taking decision [58]. In addition, it is possible that risky decisions may endure multiple mechanisms beyond disinhibition – such as “faulty cognition” and “false beliefs”, [24] sensation seeking [28] and negative mood [59]. Increased risk-taking behavior and decreased use of contextual information has been observed when affective aspects of decision-making are engaged [60].

Tattooed young women shows a preserved reflexive inhibition and interference control while express the impaired response inhibition. In current study, we aimed to assess the role of impaired inhibition control in risky decisions among tattooed persons. Although, it is likely that several of inhibition facets may contribute differentially to risky behavior all three investigated inhibition pathways not accounted for the variance in the relation between risky decision and tattooing behavior.

## Conclusions

This study defined the behavioral and impulse-dimension attributes of young women with tattoos. The strengths of the present study include the evaluation of a population of young women with limited number of tattoos, absence of: criminal or antisocial behavior, unemployment and substance use disorders, as well as using standard cognitive performance tasks that compared risk-taking decisions with different inhibition-related tasking challenges. The major limitation of the present study is the inclusion of healthy population of women only, which confers the advantage of relative homogeneity, but limits the generalizability to the general tattooed population. Unfortunately, only three inhibition paradigms were used in this study. More measures/domains of inhibition (e.g. reward paradigm of inhibition, waiting ability, “interruptive inhibition” of ongoing responses) should be assessed in an attempt to clarify the association between inhibition control and risky decision in tattooed women.

This study was designed to clarify the associations between inhibition ability and decision making process among young tattooed women. Inhibition capacities of tattooed women are preserved except slowness in the Go/NoGo task. Thus, it appears that risky decision in tattooed women is not a direct consequence of impaired inhibition capacities**.**

It may be concluded that risk-taking decisions as assessed in the IGT (hot EF) and inhibition abilities (cool EF) are different and independent aspects of self-control, as was suggested in previous studies in non-tattooed population [26–31, 41]. It should be noted that in the absence of a cutoff value for the IGT performance, the current analysis shows that the tattooed group had a significantly lower score than non-tattooed women (“group differences”), but the results cannot indicate an “impairment” in decision-making.

The limited research in tattooing persons, justifies further studies that will explore the multiple aspects of possible association between inhibition capacities and risky decisions, including variables such as the number of tattoos, their size and body localization, as well as their psychological meaning.

## Data Availability

The datasets used and/or analysed during the current study are available from the corresponding author on reasonable request.

## Abbreviations

AK: Alexandr Kagan
AW: Abraham Weizman
IGT: Iowa Gambling Test
MFFT: Matched Familiar Figure Test
OH: Omer Hegedish
RL: Rina Lapidus
SK: Semion Kertzman
